# Supplementary material for: Intelligent Identification of MoS2 Nanostructures with Hyperspectral Imaging by 3D-CNN
Source: Nanomaterials (Basel). 2020 Jun 13;10(6):1161. doi: 10.3390/nano10061161 (PMC7353172; doi:10.3390/nano10061161)
Supplement: Supplementary file 1 [file nanomaterials-10-01161-s001.pdf]

nanomaterials

Supplementary Materials

Intelligent Identification of MoS_2 Nanostructures with Hyperspectral Imaging by 3D-CNN

Kai-Chun Li [1], Ming-Yen Lu [2], Hong Thai Nguyen [1], Shih-Wei Feng [3], Sofya B. Artemkina [4,5], Vladimir E. Fedorov [4,5] and Hsiang-Chen Wang [1,*]

1 Department of Mechanical Engineering and Advanced Institute of Manufacturing with High tech Innovations, National Chung Cheng University, 168, University Rd., Min Hsiung, Chia Yi 62102, Taiwan; zuoo549674@gmail.com (K.-C.L.); nguyenhongthai194@gmail.com (H.T.N.)
2 Department of Materials Science and Engineering, National Tsing Hua University, 101, Sec. 2, Kuang-Fu Road, Hsinchu 30013, Taiwan; mingyenlu@gmail.com
3 Department of Applied Physics, National University of Kaohsiung, 700 Kaohsiung University Rd., Nanzih District, Kaohsiung 81148, Taiwan; swfeng@nuk.edu.tw
4 Nikolaev Institute of Inorganic Chemistry, Siberian Branch of Russian Academy of Sciences, Novosibirsk 630090, Russia; artem@niic.nsc.ru (S.B.A.); fed@niic.nsc.ru (V.E.F.)
5 Department of Natural Sciences, Novosibirsk State University, 1, Pirogova str., Novosibirsk 630090, Russia
* Correspondence: hcwang@ccu.edu.tw

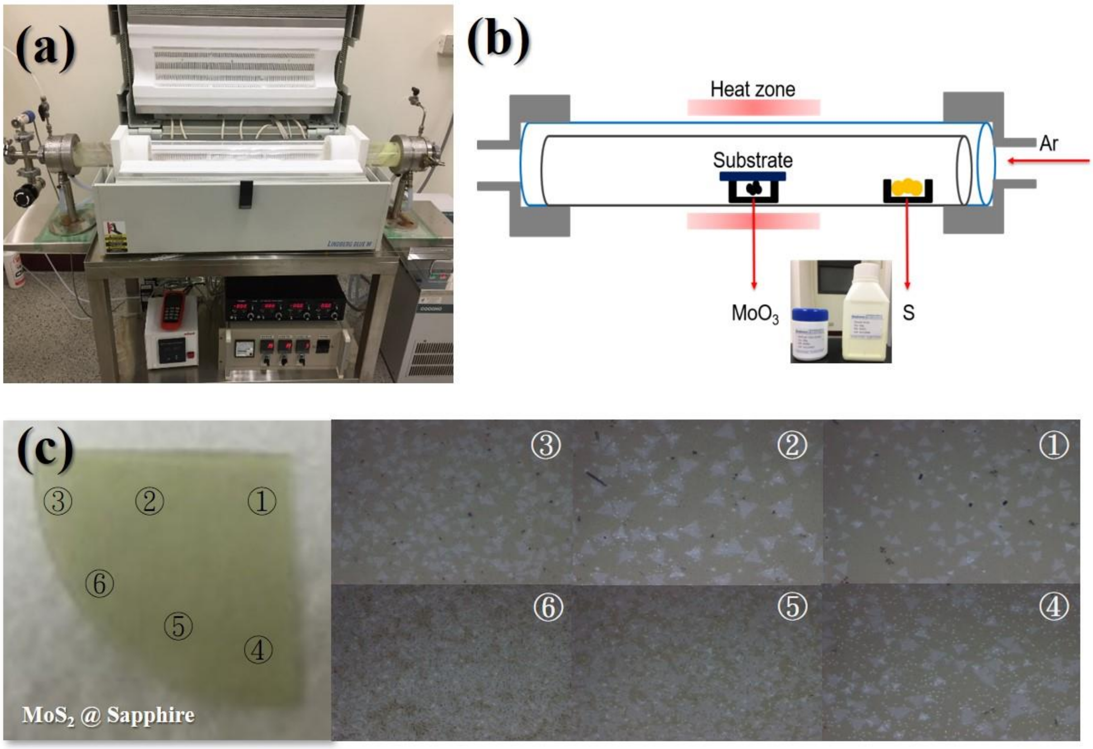

Figure S1. Experimental device. (**a**) Three-zone furnace tube used in this experiment (Lindberg/Blue HTF55347C), (**b**) schematic of the experimental structure and position of the precursor, (**c**) Area-coded position and image of the sample under OM shooting.

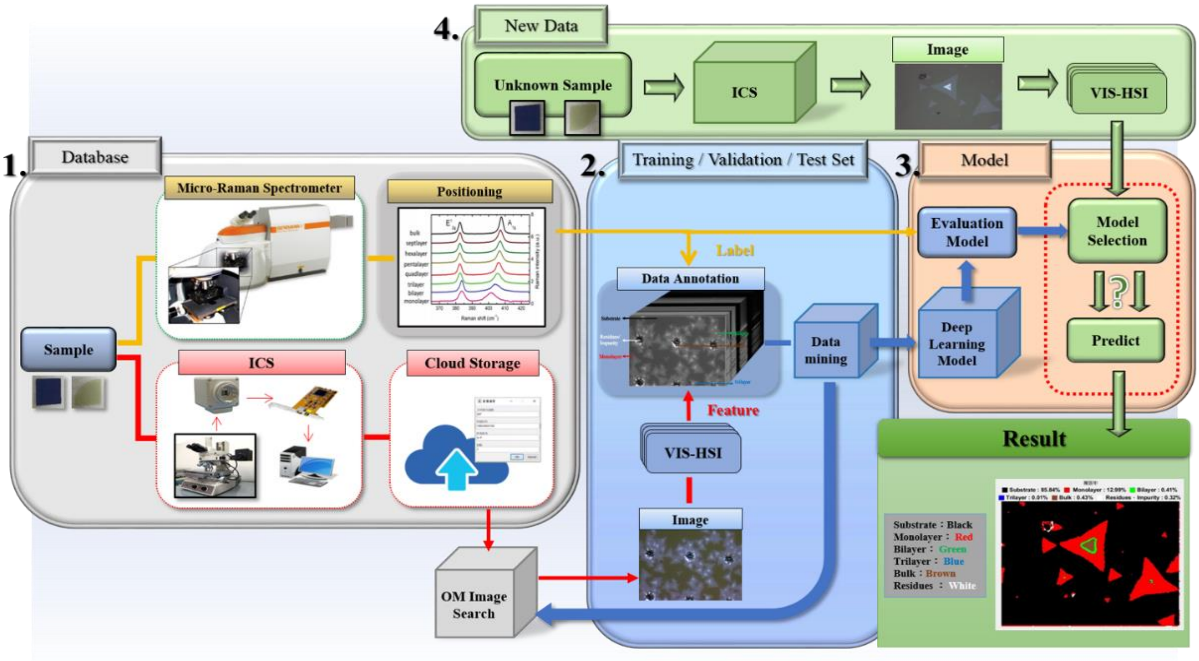

Figure S2. Deep learning is applied to construct a flowchart for the number detection system of the optical MoS_2 layer. The gray, blue, orange, and green block colors correspond to (1) database, (2) offline training, (3) model design, and (4) online service, respectively.

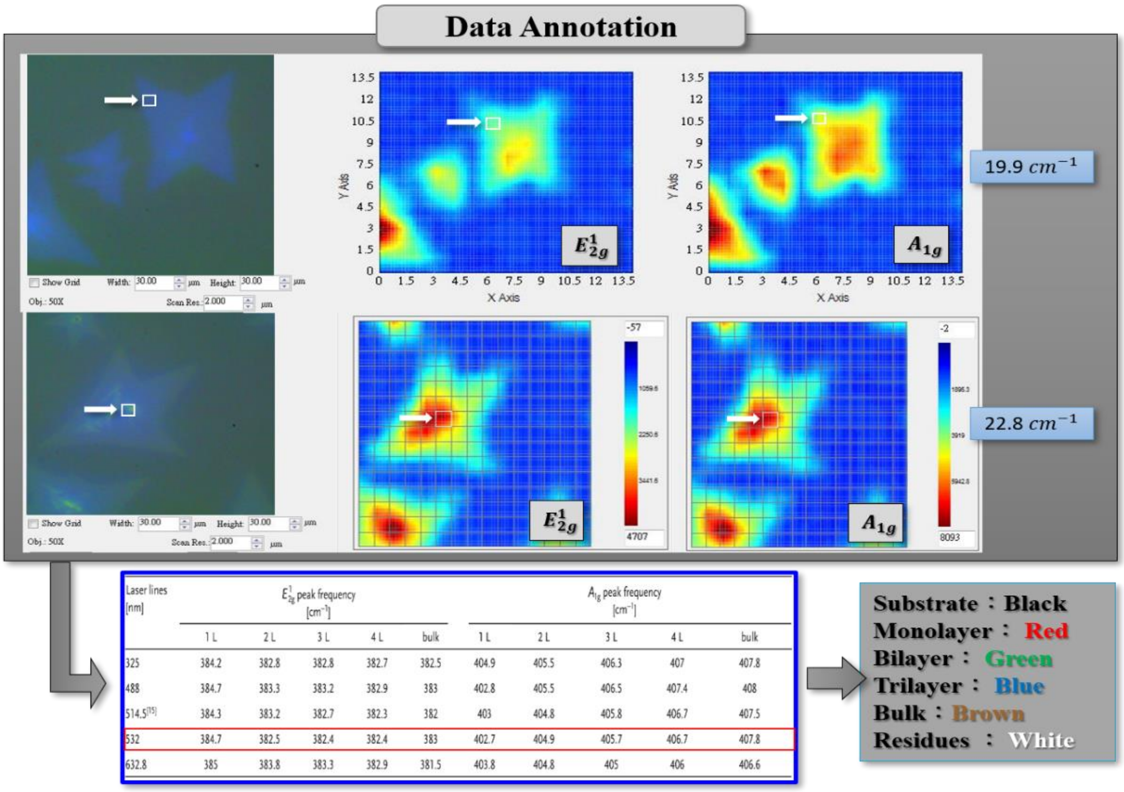

Laser lines [nm]	E^1_{2g} peak frequency [cm^{-1}]					A_{1g} peak frequency [cm^{-1}]				
	1 L	2 L	3 L	4 L	bulk	1 L	2 L	3 L	4 L	bulk
325	384.2	382.8	382.8	382.7	382.5	404.9	405.5	406.3	407	407.8
488	384.7	383.3	383.2	382.9	383	402.8	405.5	406.5	407.4	408
514.5[15]	384.3	383.2	382.7	382.3	382	403	404.8	405.8	406.7	407.5
532	384.7	382.5	382.4	382.4	383	402.7	404.9	405.7	406.7	407.8
632.8	385	383.8	383.3	382.9	381.5	403.8	404.8	405	406	406.6

Figure S3. Data labeling is assisted by the Raman spectroscopy instrument (MRI-1532A). This instrument is mainly used to inject a 532 nm laser light into the sample. The photons in the laser will collide with the molecules in the sample material, namely, E^1_{2g} and A_{1g}. The peak difference of the vibration mode is the signal of the main judgment layer of MoS2, and the two vibration modes have a high dependence on the thickness of MoS_2. We select two 30 μm×30 μm Raman mapping results in the database and wait for ~45 min, especially in the ground truth mark, which is a considerable time.

Visible Hyperspectral Imaging Algorithm

We use 24 color blocks as a common target and obtain corresponding RGB values and spectral data through cameras and spectrum analyzers. As the camera processes CCD through image signal processing and 3A (autofocus [AF], autoexposure [AE], and auto white balance [AWB]), the camera image must be corrected to the actual physical phenomenon. The presented analysis is modified by converting the RGB values and spectral data into an XYZ gamut space and performing color-adaptive transformation and multivariate linear regression analysis to obtain a camera correction matrix. The spectral data are then subjected to principal component analysis, and the first twelve groups of principal components are compared with the corrected XYZ values for multivariate linear regression analysis. Finally, the transformation matrix is obtained.

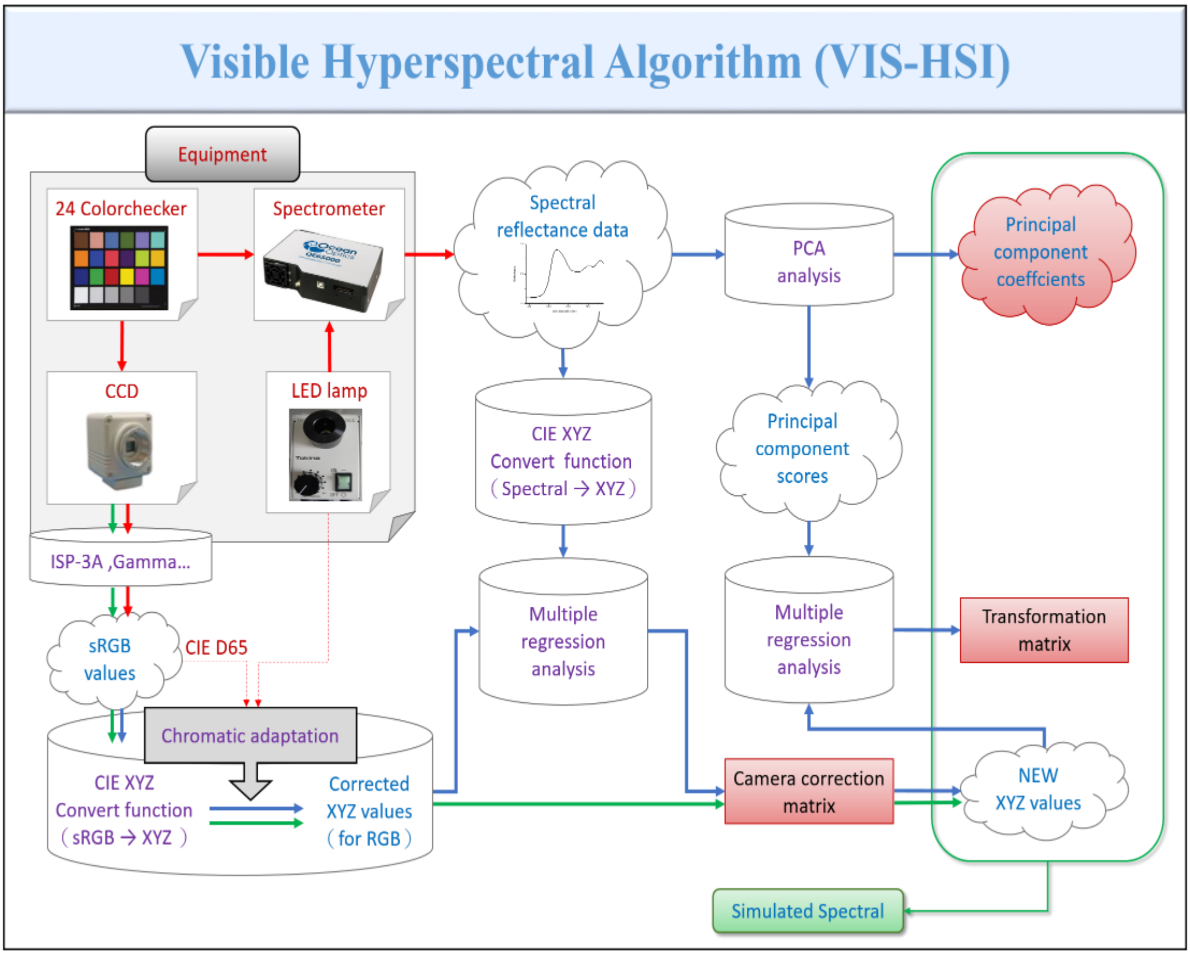

Figure S4. Flowchart of visible hyperspectral image algorithm.

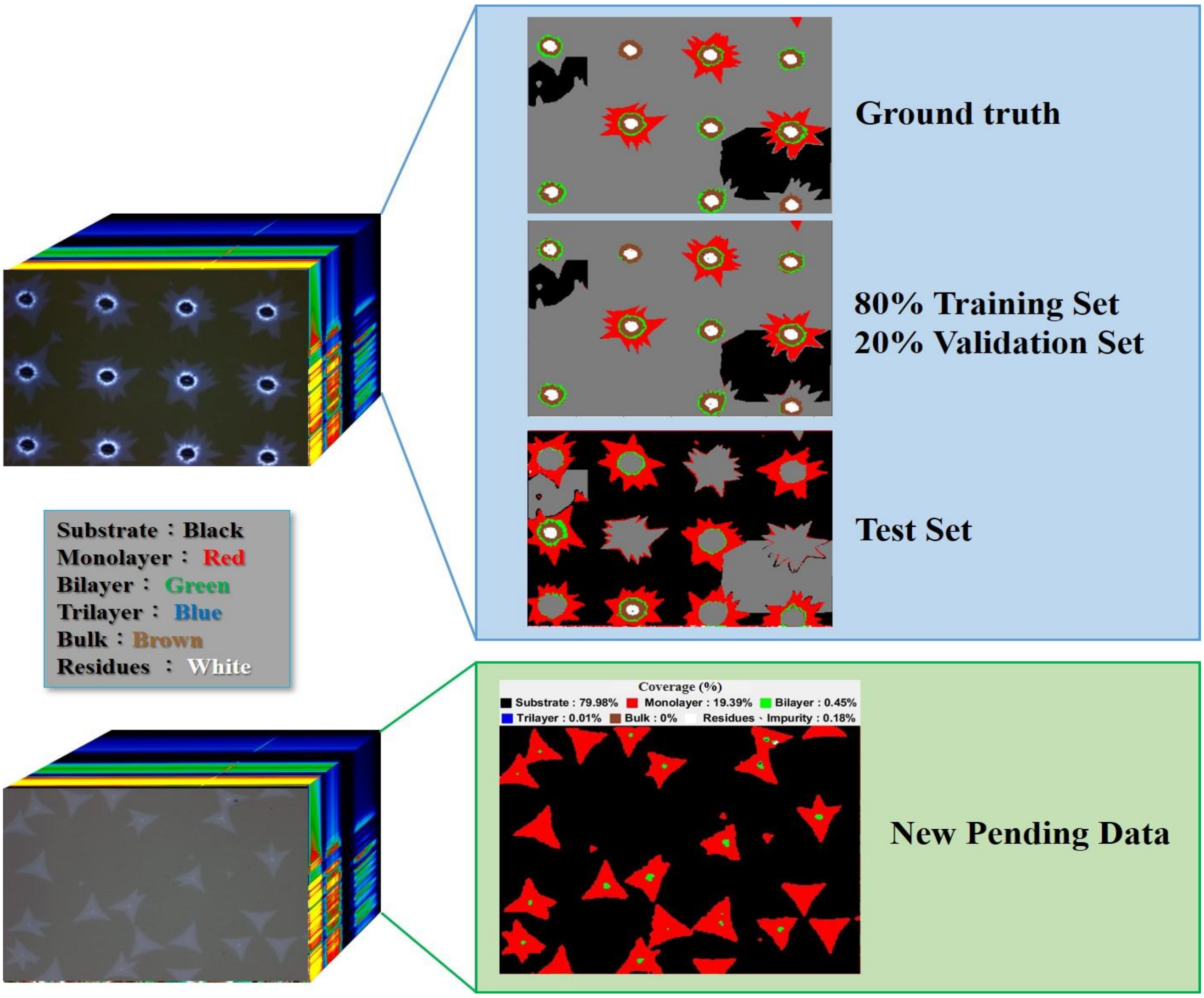

Figure S5. In the blue area, the data in the offline training section of Figure 2 are used. The ground truth and our label data are set as the training and validation sets, respectively. The rest of the VIS-HIS feature data are used as the test set. The green area is the predicted result of new pending data in the (4) online service architecture in Figure S2.

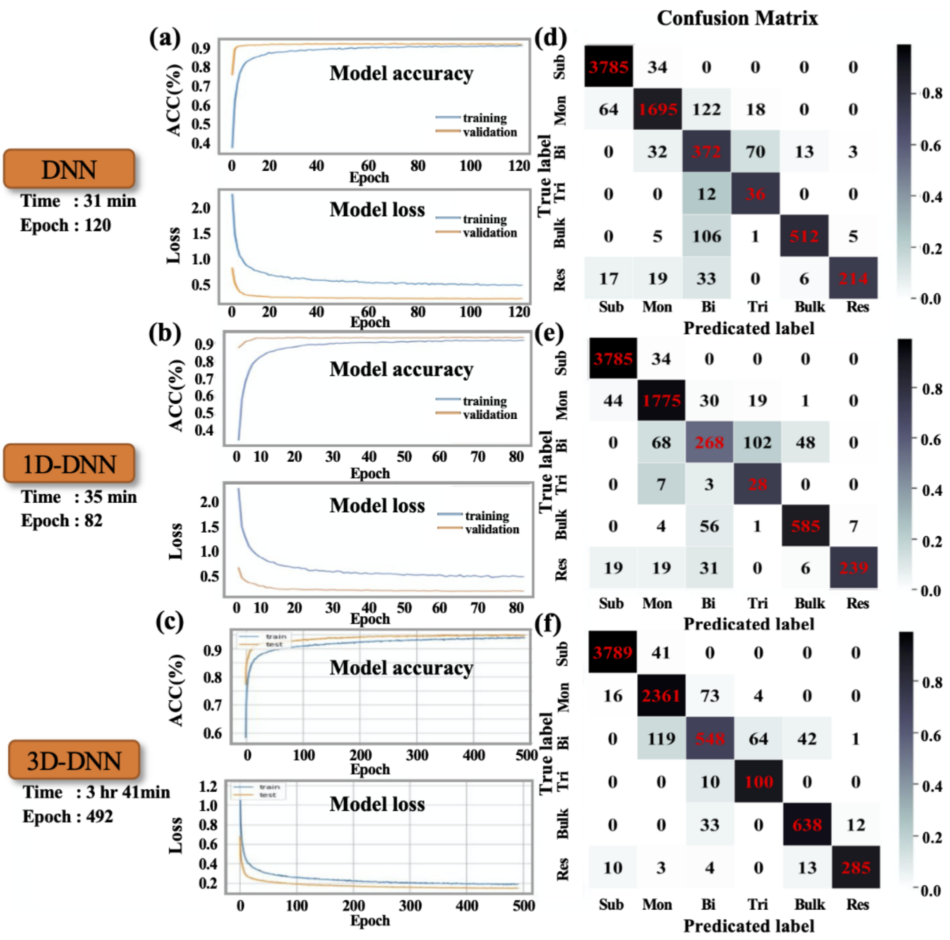

Figure S6. At 40× magnification, the accuracy (ACC) and loss of the convergence process in (**a**) DNN, (**b**) 1D-CNN, and (**c**) 3D-CNN; confusion matrix results of the validation set in (**d**) DNN, (**e**) 1D-CNN, and (**f**) 3D-CNN.

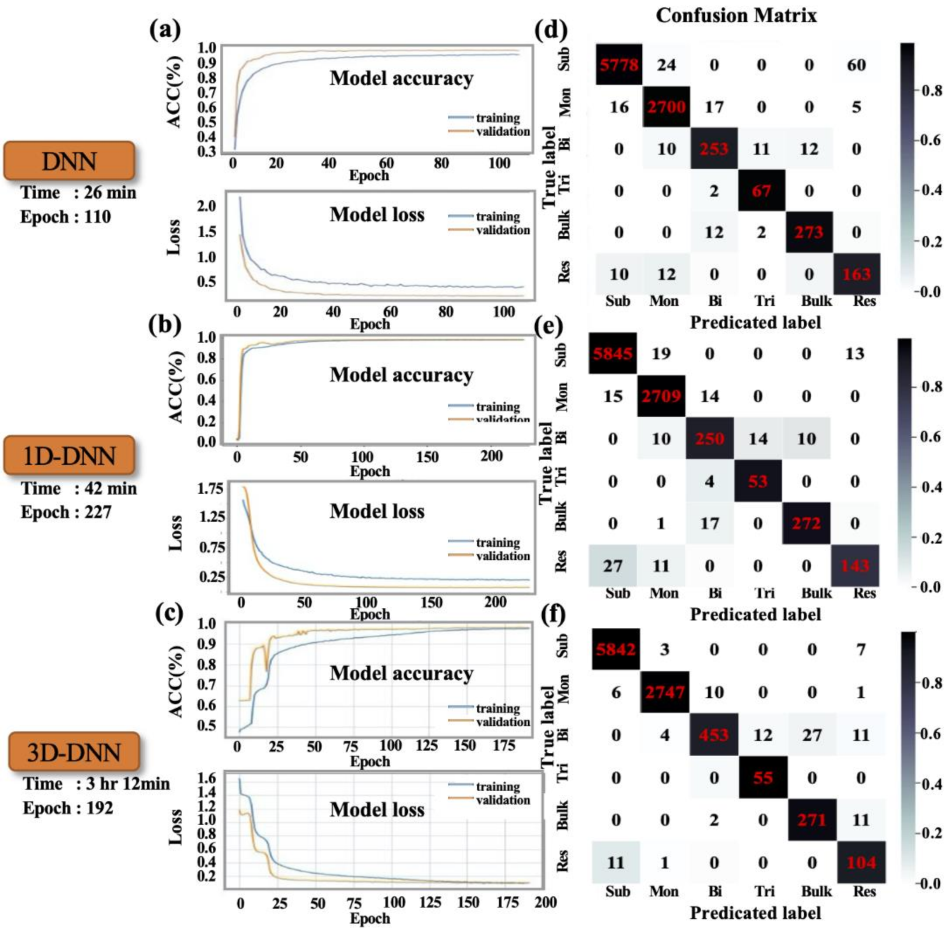

Figure S7. At 100× magnification, the accuracy (ACC) and loss of the convergence process in (**a**) DNN, (**b**) 1D-CNN, and (**c**) 3D-CNN; confusion matrix results of the validation set in (**d**) DNN, (**e**) 1D-CNN, and (**f**) 3D-CNN.

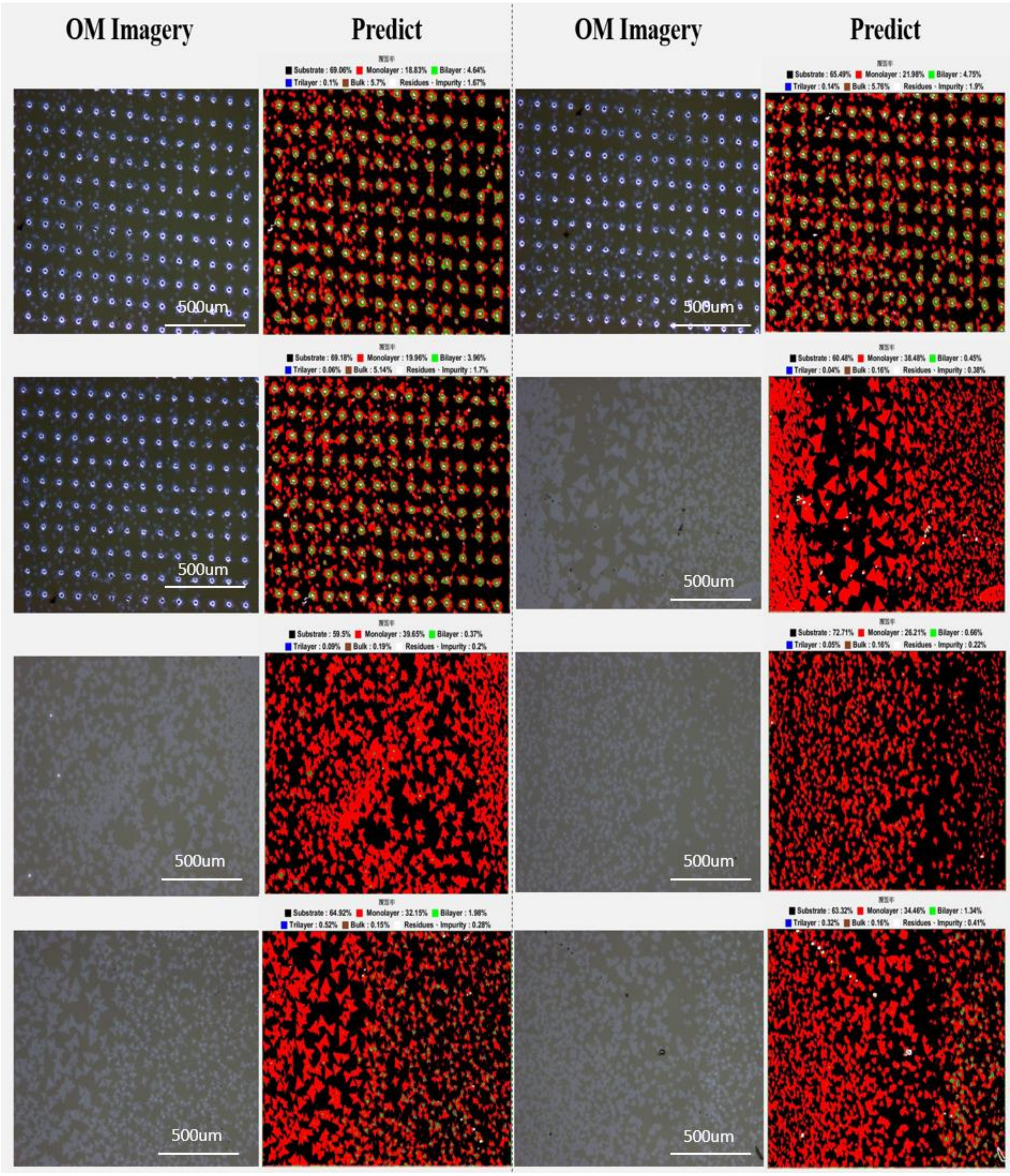

Figure S8. OM image and prediction results of the new pending data at 10× magnification.

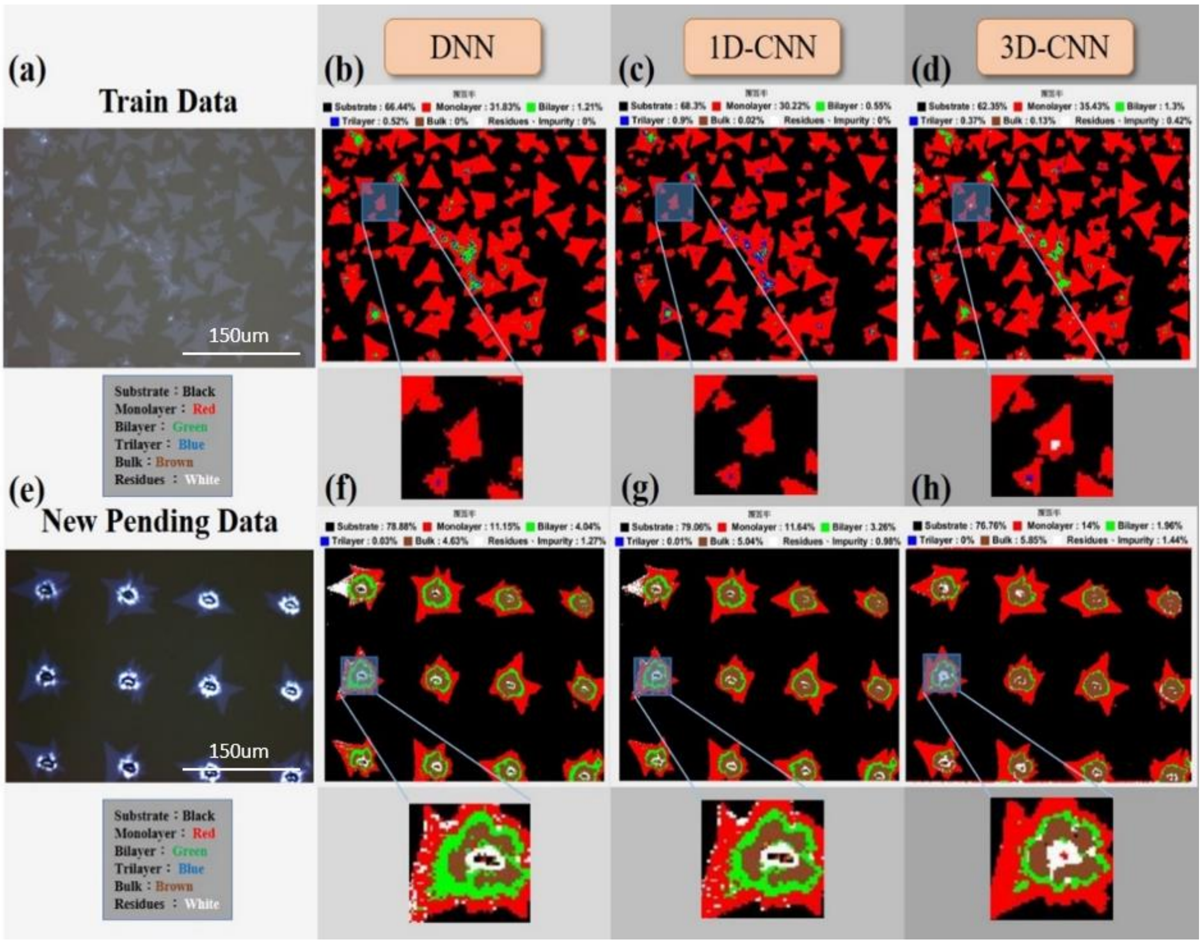

Figure S9. At 40× magnification, (**a**) and (**e**) are the OM images of train data and new pending data, respectively. The predicted results of the three models are the false-color composite predicted by (**b**) DNN, (**c**) 1D-CNN, and (**d**) 3D-CNN in the training data, and DNN and 1D-CNN can be observed. In the CNN, impurities in the single layer are undetected. New pending data predict the color classification image (false-color composite) by (**f**) DNN, (**g**) 1D-CNN, and (**h**) 3D-CNN and can observe errors in a single layer in DNN and 1D-CNN. The result of misclassification of impurity classification.

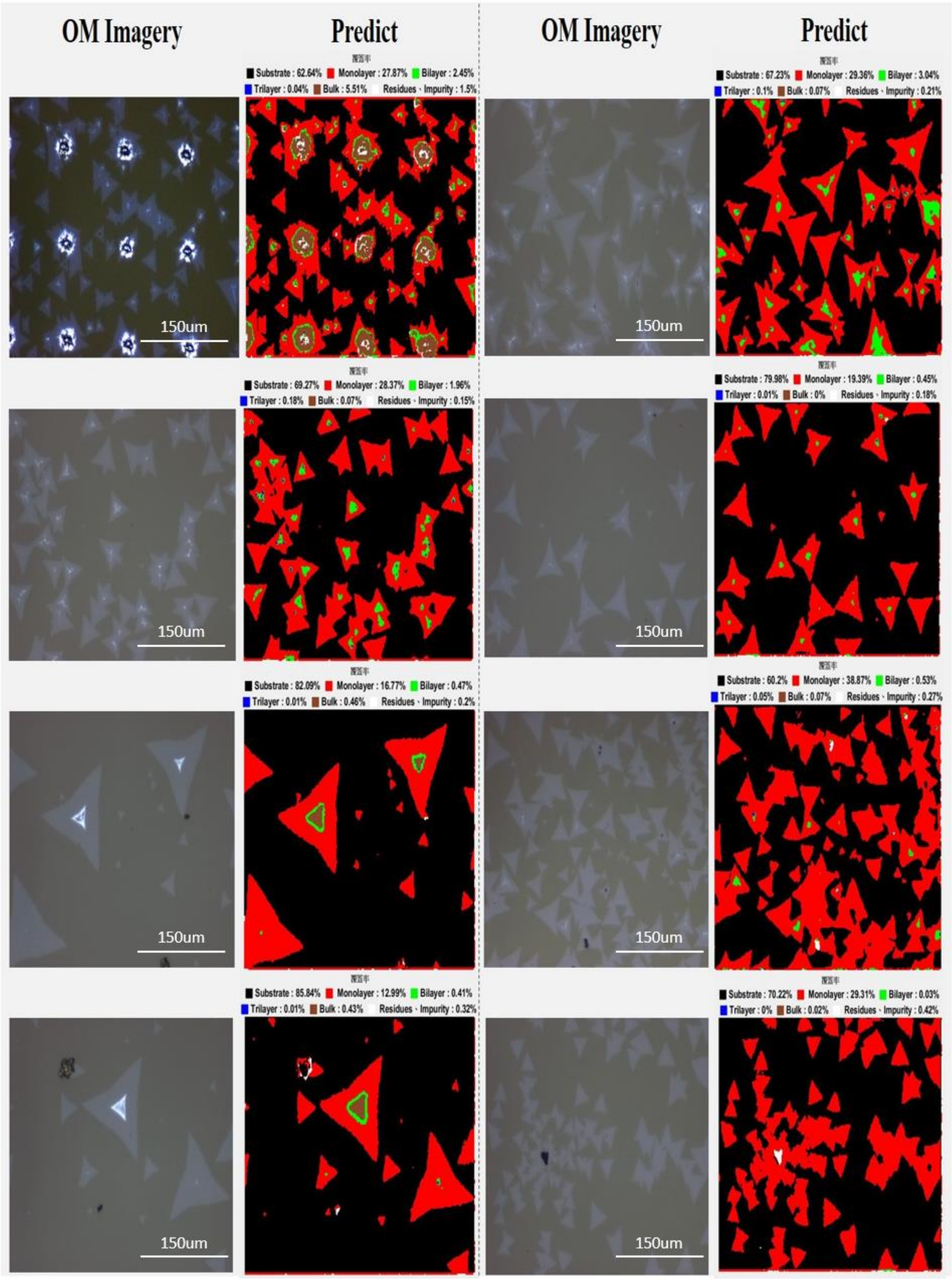

Figure S10. OM image and prediction results of the new pending data at 40× magnification.

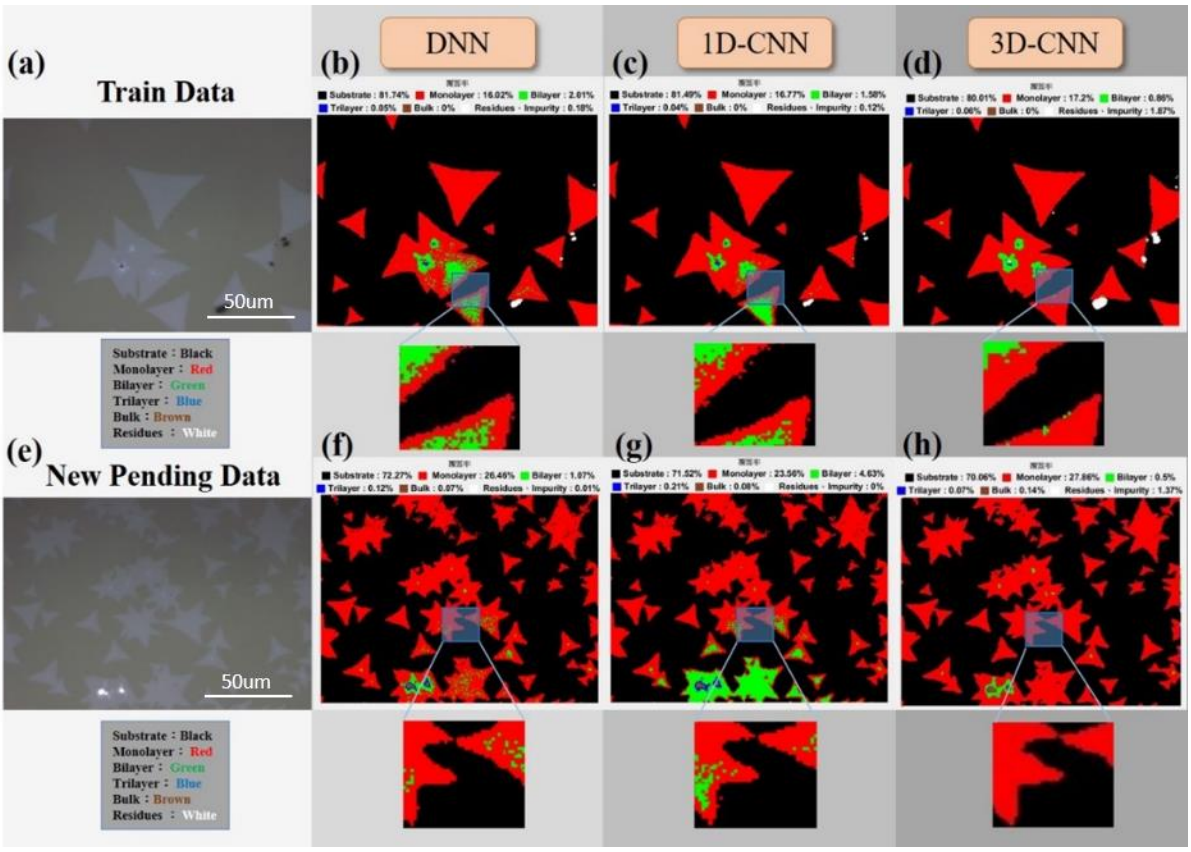

Figure S11. At 100× magnification, (**a**) and (**e**) are the OM images of the training (train data) and new test (new pending data) sets. (**b**) DNN, (**c**) 1D-CNN, and (**d**) 3D-CNN indicate the prediction results of the color classification image (false-color composite) in the training data. (**f**) DNN, (**g**) 1D-CNN, and (**h**) 3D-CNN reflect the prediction results of the color classification image (false-color composite) in the new pending data, from DNN and 1D-CNN in the single layer with the wrong double-layer classification misjudgment results.

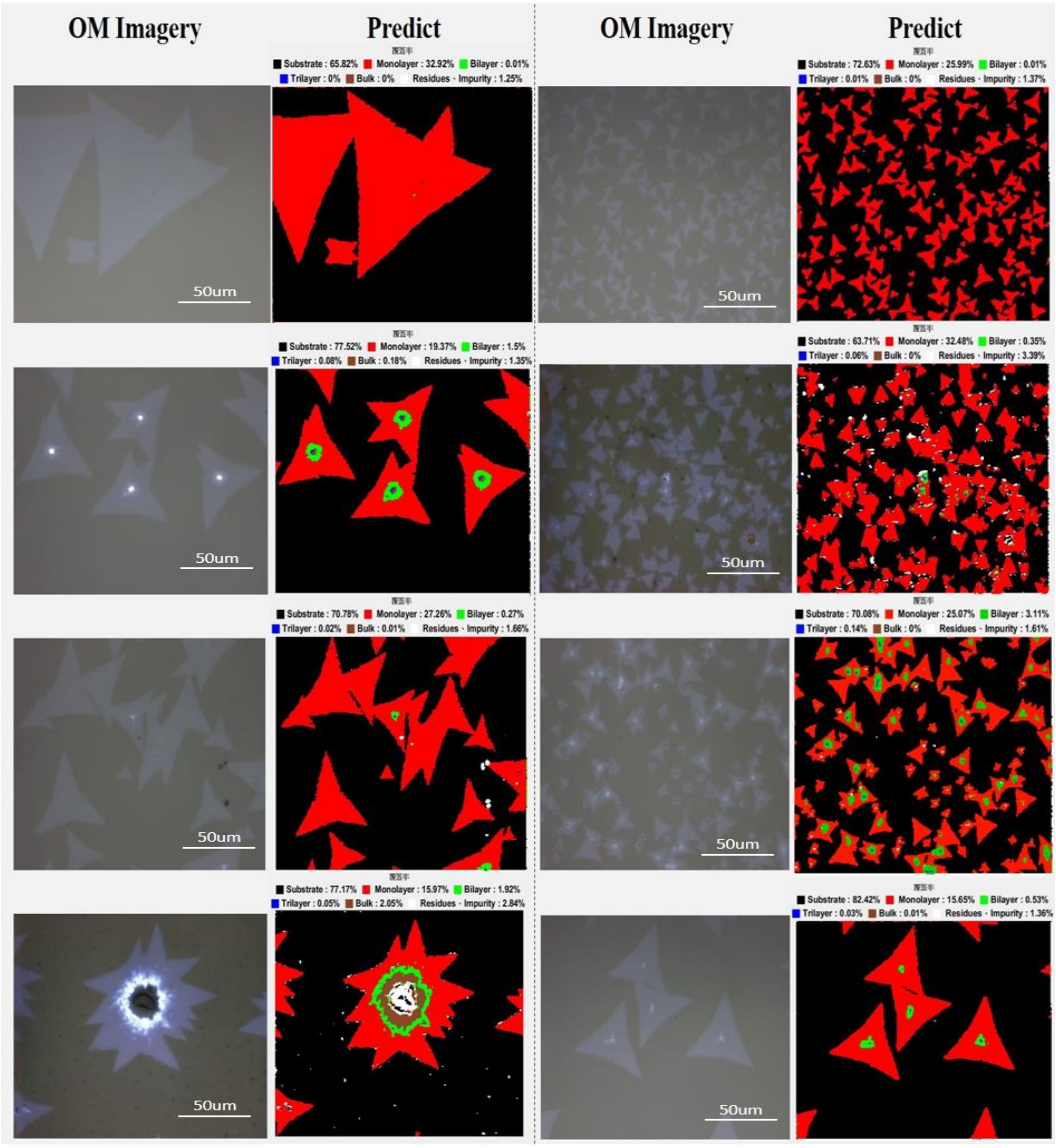

Figure S12. OM image and prediction results of the new pending data at 100× magnification.

Distribution of Light Intensity in Angular Illumination Asymmetry

Initially, we only take OM images of sapphire substrates (Figures S13a,d,g) for three different magnification rates of 10×, 40×, and 100×. Then, we convert the RGB color space of the OM image to the HSV color space. As the RGB channels are related to the color brightness, the color information cannot be effectively separated from the brightness. From the color science-related course, the HSV is suitable for image brightness and the separation of the color information, as shown in Figures S13b,e,h). We obtain the V channel (lightness) to express the light intensity distribution of the image. Then, we further observe from the RGB scatter plot that the uneven light intensity pixels are roughly linearly distributed, as shown in Figures S13c,f,i. Thus, we use singular value decomposition for linearity. The distributed pixels are linearly fitted. This step is intended to generalize the color space of the OM image of the sapphire substrate within a defined linear range.

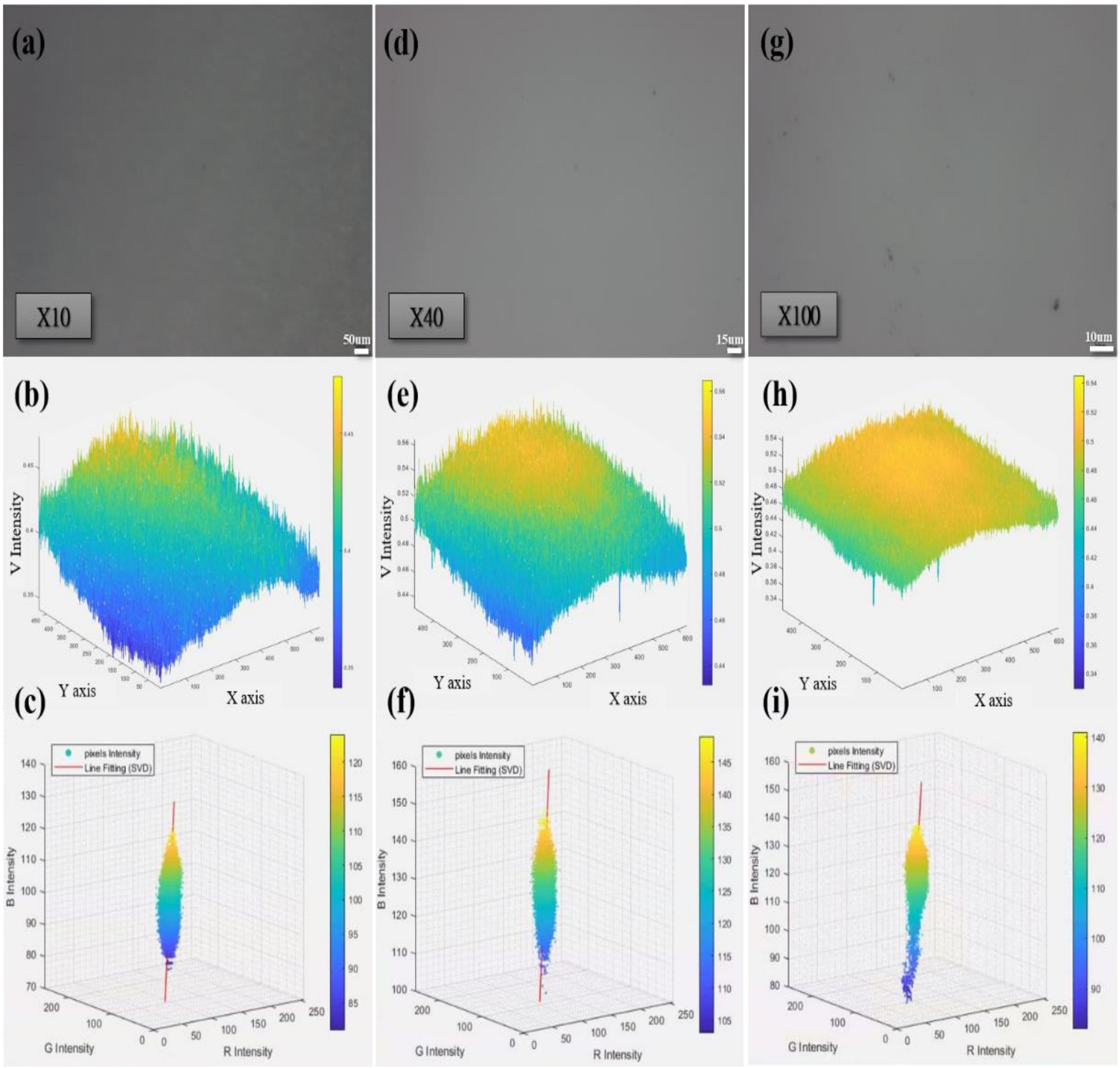

Figure S13. Optical microscope light intensity distribution analysis. (**a**,**d**,**g**) OM images of sapphire substrate taken at 10×, 40×, and 100× magnification rates (50, 15, and 10 μm in the lower right corner, respectively). (**b**,**e**,**h**) V channels in the HSV color space of the microscope image (lightness) light intensity distribution. (**c**,**f**,**i**) Scatter plots of each pixel point RGB channel in the OM image.

Analysis of the Prediction Results of Laser Drilling Substrate Pretreatment

The color classification image can only display the final classification result. We cannot determine the index of the model's confidence in the prediction of each classification layer and category. Therefore, we visually observe the prediction probability between each classification category, as shown in Figures S14a–c. The substrate, single layer, double layer, triple layer, multilayer and pollutant/impurity prediction probability distribution of one of the 10×, 40×, and 100× magnification training databases are presented. The confidence value predicted for each classification type is maintained at a certain level, which can explain the fact that the micro-state features are blurred or the impurities are further difficult to identify because the prediction probability is low, but the phenomenon is mainly caused by spatial resolution.

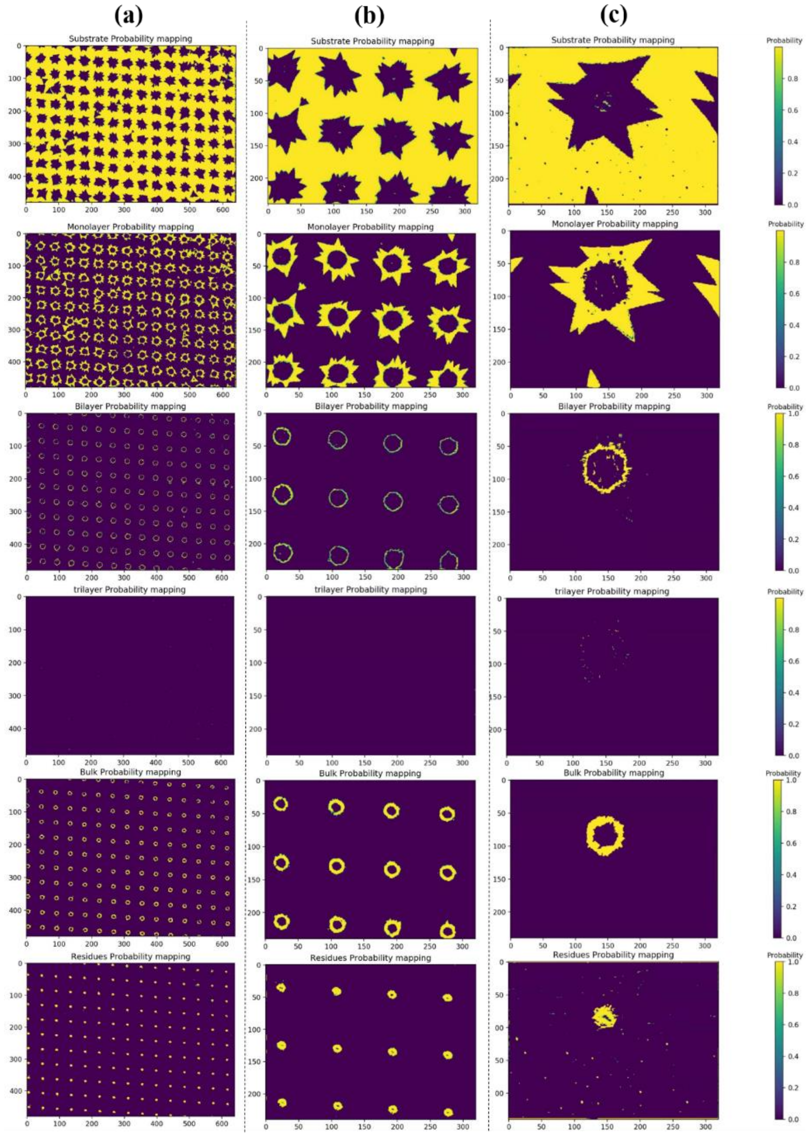

Figure S14. Prediction rate of each classification type at the shooting magnification rates of (**a**) 10×, (**b**) 40×, and (**c**) 100×.

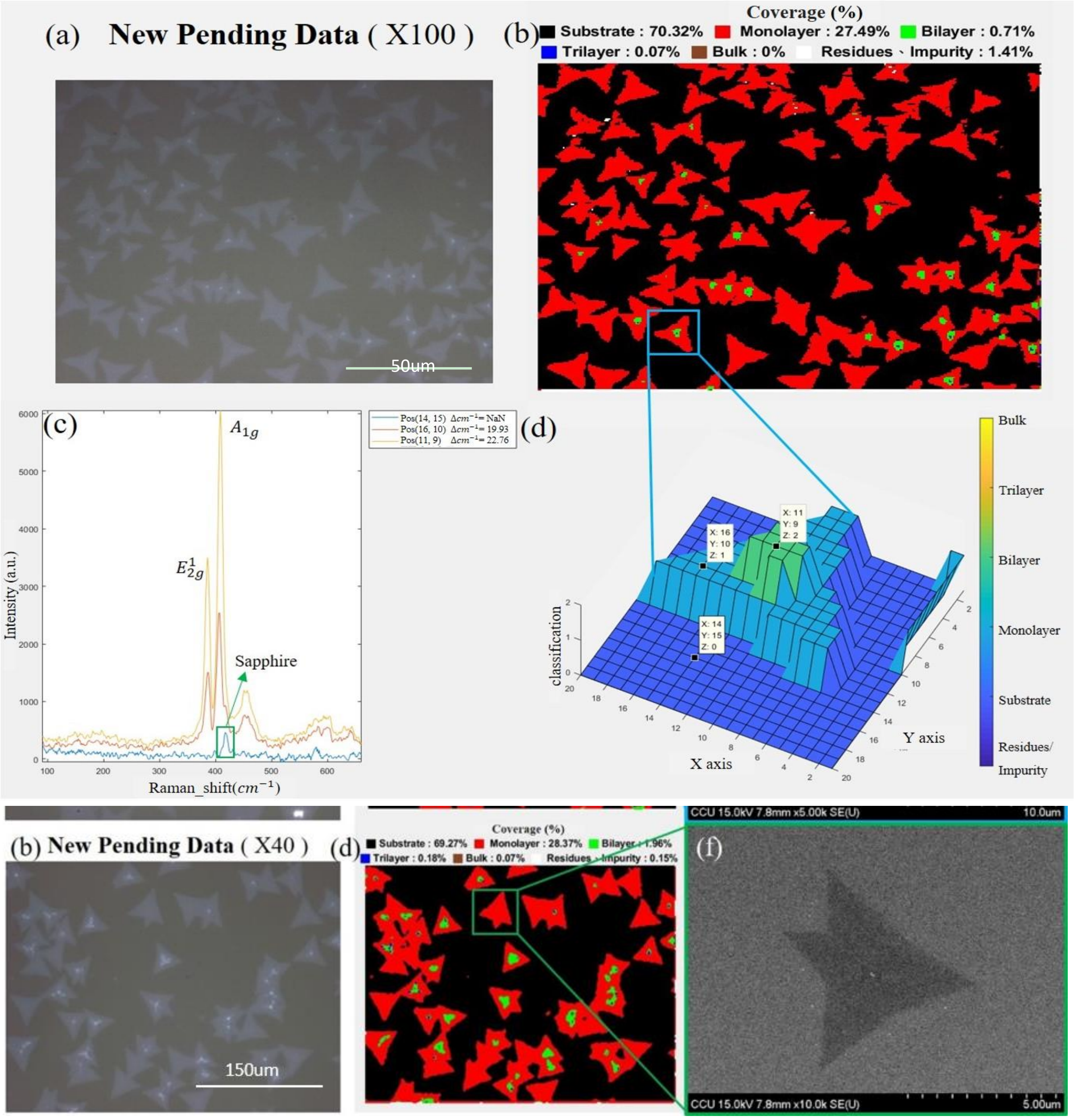

Figure S16. SEM measurement of the hyperspectral image of the new test data (new data). (**a**,**b**) are OM images at 100× and 40× magnification rates, (c, d) are prediction results corresponding to the (**a**,**b**) OM image ranges, and (**e**,**f**) correspond to (**c**,**d**) ROI measurement results.

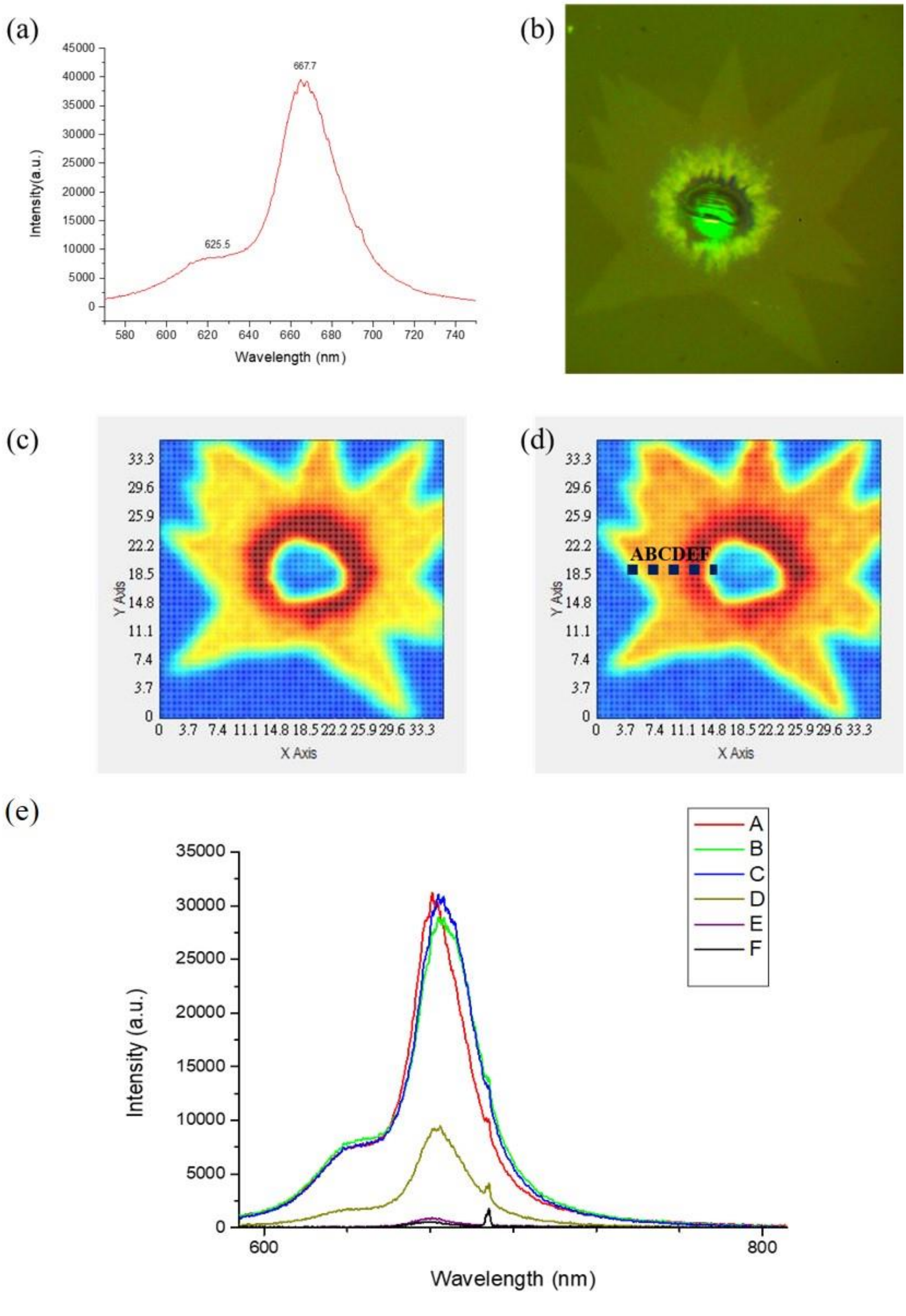

Figure S17. (**a**) Photoluminescence spectrometry. (**b**) OM image of selected PL mapping range. (**c**) PL mapping with a wavelength of 625 nm (**d**) PL mapping with a wavelength of 667 nm. (**e**) The blue measurement in (**d**) indicates the PL measurement result of the test piece.

Analysis of the Growth of MoS_2 through Laser Borehole Measurement by HRTEM

Figure S18a shows the OM image after growing MoS_2. The red line marks the FIB sampling position, and Figure S18b presents the cross-sectional TEM image of the selected area of Figure S18a. Figure S18c displays a magnified TEM image at the red arrow in Figure S18b. The box portion is magnified at a high rate and converted to SAED. Figure S18d exhibits the red box in Figure S18c. At the HRTEM image, the position is the growth of the multilayer MoS_2. Figure S18e shows the HRTEM image of the orange box at Figure S18c. The sampling position is in the mixture layer. Figure S18f presents the yellow box of Figure S18c. At the HRTEM image, the position is selected at the junction of the mixed area and sapphire substrate. The two-layer MoS_2 grows clearly in the image. Figure S18g displays the SAED diagram of Figure S18d. The angle of view is [001]. The lattice array of the multilayer MoS_2 can be seen. On the basis of the distance between the layers, the hexagonal crystal structure of MoS_2, and the lattice constant, a = 0.318 nm, c = 1.299 nm, and the plane distance between the MoS_2 layers is 6.2 Å, which is close to that of the 2-H MoS_2 crystal plane (6.5 Å) [65], as shown in Figure S18h. In the SAED diagram Figure S18e, the angle of the photograph is [001], and its lattice arrangement is confusing, but the lattice points looming in four directions may be an array of MoO_3 lattices [64]. Figure S18i shows the SAED diagram of Figure S18f. The viewing angle is still [001], and the selected region is the intersection of the mixed region and sapphire substrate at the double layer of the HRTEM, because the lattice at the boundary may be around the boundary of the boundary. However, from the center point, the angle of view of [001] can be obtained and the crystal plane distance of approximately 6.4 Å can be calculated [65]

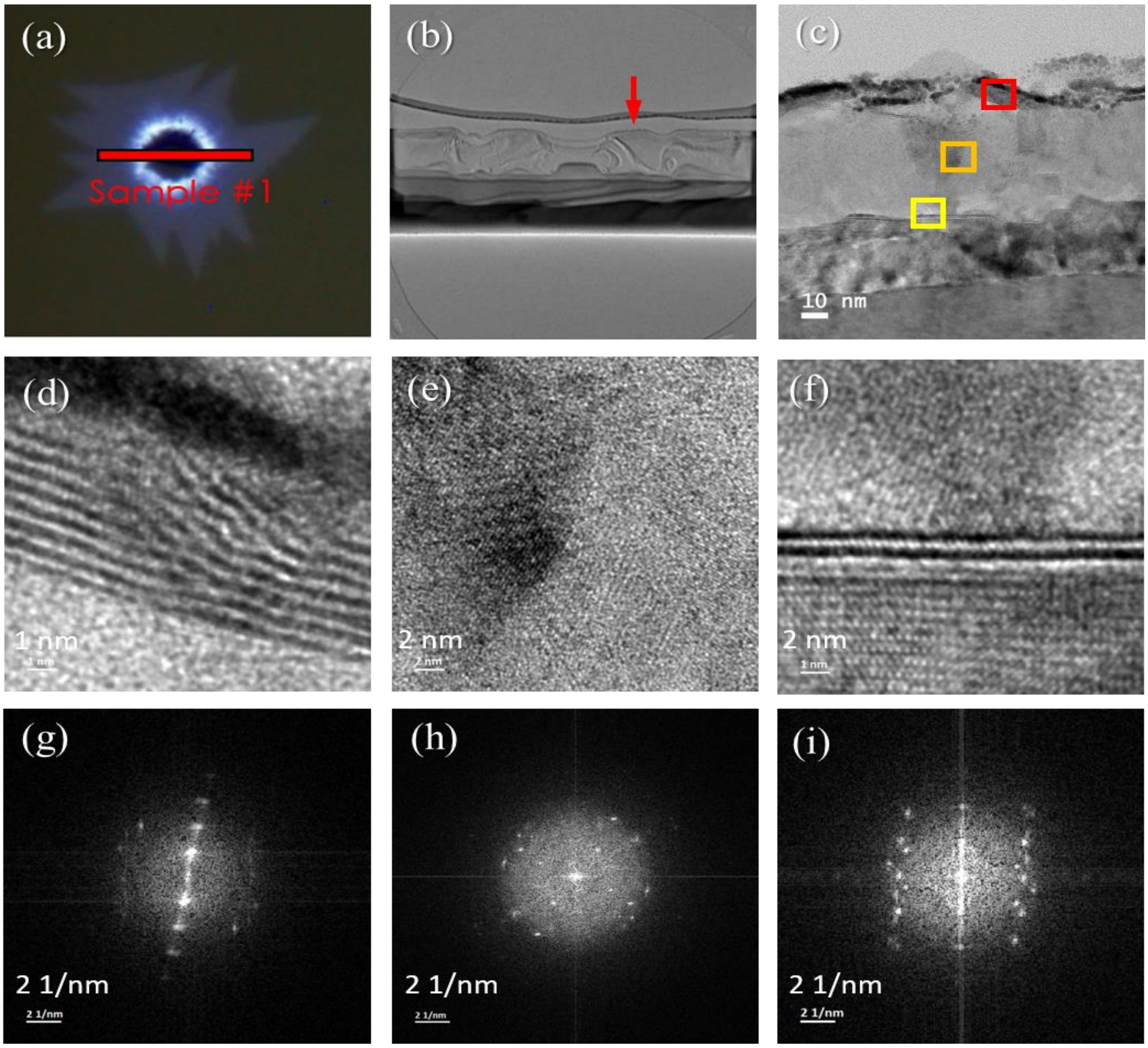

Figure S18. (**a**) is the OM image after growing MoS_2, (**b**) is the cross-sectional TEM image of the selected area of (**a**), (**c**) is the magnified TEM image of the red arrow of (**b**). (**d**) is the HRTEM image at the red box in (**c**). (**e**) is the HRTEM image at the orange box in (**c**), and (**f**) is the HRTEM image at the yellow box in (**c**). (g) is the SAED diagram of (**d**), (**h**) is the SAED diagram of (**e**), and (**i**) is the SAED diagram of (**f**).
